# Predicting the Impact of Climate Change on the Habitat Distribution of *Parthenium hysterophorus* around the World and in South Korea

**DOI:** 10.3390/biology12010084

**Published:** 2023-01-04

**Authors:** Pradeep Adhikari, Yong-Ho Lee, Anil Poudel, Gaeun Lee, Sun-Hee Hong, Yong-Soon Park

**Affiliations:** 1 Institute of Humanities and Ecology Consensus Resilience Lab, Hankyong National University, Anseong 17579, Republic of Korea; 2 OJeong Resilience Institute, Korea University, Seoul 02841, Republic of Korea; 3 School of Plant Science and Landscape Architecture, College of Agriculture and Life Sciences, Hankyong National University, Anseong 17579, Republic of Korea; 4 Department of Plant Resources, College of Industrial Sciences, Kongju National University, Yesan 32439, Republic of Korea

**Keywords:** climate change, habitat suitability, invasive species, *Parthenium hysterophorus*, MaxEnt

## Abstract

**Simple Summary:**

*Parthenium hysterophorus* is one of the most noxious invasive weeds in the world. In this study, a species distribution model of *P*. *hysterophorus* was established using the maximum entropy (MaxEnt) modeling approach. According to the model, climate change would likely increase the habitat suitability for *P*. *hysterophorus* across the world. Estimation of mean habitat suitability revealed that 21 countries including Bulgaria, Brunei, China, Netherlands, New Zealand, and South Korea, currently in the low habitat suitability category, would transition into the moderate to very high suitability category by 2081–2100. In South Korea, climate change would increase the habitat suitability for *P*. *hysterophorus*, especially in the southern region of the country, which would be followed by spontaneous expansion towards the northern region, thus seriously threatening agriculture, native biodiversity, ecosystem services, and the national economy. Therefore, regular monitoring is required to perform to prevent further habitat expansion of *P*. *hysterophorus* in South Korea.

**Abstract:**

The global climate change, including increases in temperature and precipitation, may exacerbate the invasion by *P*. *hysterophorus*. Here, MaxEnt modeling was performed to predict *P*. *hysterophorus* distribution worldwide and in South Korea under the current and future climate global climate changes, including increases in temperature and precipitation. Under the current climate, *P*. *hysterophorus* was estimated to occupy 91.26%, 83.26%, and 62.75% of the total land area of Australia, South America, and Oceania, respectively. However, under future climate scenarios, the habitat distribution of *P*. *hysterophorus* would show the greatest change in Europe (56.65%) and would extend up to 65°N by 2081–2100 in South Korea, *P*. *hysterophorus* currently potentially colonizing 2.24% of the land area, particularly in six administrative divisions. In the future, *P*. *hysterophorus* would spread rapidly, colonizing all administrative divisions, except Incheon, by 2081–2100. Additionally, the southern and central regions of South Korea showed greater habitat suitability than the northern region. These findings suggest that future climate change will increase *P*. *hysterophorus* distribution both globally and locally. Therefore, effective control and management strategies should be employed around the world and in South Korea to restrict the habitat expansion of *P*. *hysterophorus*.

## 1. Introduction

*Parthenium hysterophorus*, a prolific invasive species, is native to the tropical and subtropical regions of America [1]. *P. hysterophorus* is an annual herbaceous plant species belonging to the family *Asteraceae* [2] and is recognized as one of the 100 of the world’s worst invasive species. *P. hysterophorus* exhibits high fecundity and rapid germination and growth and produces a number of growth-inhibiting phytochemicals that suppress the germination and growth of the surrounding flora [2,3,4]. Moreover, it is highly competitive with pasture plants and food crops for soil nutrients (e.g., nitrogen and soil moisture) [5].

*P. hysterophorus* can tolerate salt and drought stresses [6] and usually invades disturbed land, but it can adapt to a variety of habitats including croplands, pastures, orchards, and the edges of forests [1], which makes it one of the most noxious invasive weeds in the world, threatening biodiversity and significantly reducing crop and pasture yield [2]. In India, *P. hysterophorus* has been reported to decrease crop and fodder yield by up to 63% and 90%, respectively [7]. Moreover, *P. hysterophorus* also has socioeconomic impacts, such as poisoning in livestock and wildlife, and causing pollen allergy, contact dermatitis, asthma, bronchitis, and hay fever in human beings [4,8].

The first taxonomic record of *P. hysterophorus* was in east Mexico; however, its native range is considered to span the West Indies and the adjoining coastal areas of North and South America [9]. Because of accelerated human mobility, owing to international trade and tourism and technological advancements, *P. hysterophorus* has moved into other countries of the Americas, and subsequently into Asia, Africa, Australia Oceania, and Europe [2,9]. Currently, *P. hysterophorus* is found in over 50 countries [4]. In South Korea, *P. hysterophorus* was first recorded before 1996 in Masan City, which is located along the southeastern coast of the country [10].

*P. hysterophorus* can tolerate a wide range of environments, including high temperature, extreme soil moisture, and increasing carbon dioxide (CO_2_) concentration [11], suggesting that *P. hysterophorus* is a highly prolific plant under climate change. In addition, changes in temperature and precipitation are very critical climatic factors that determine plant distribution and invasiveness [12]. Besides the climatic factors, other environmental components, such as wind, surface runoff, movement of livestock or wildlife, and anthropogenic disturbance, e.g., construction of roads, railways, and parks, influence the distribution of *P. hysterophorus* [2,3]. *P. hysterophorus* prefers neutral to alkaline pH soils, but it can grow in all types of soils in fields and wastelands [13]. Therefore, to manage invasive species, conserve native biodiversity, and understand the establishment and spread of invasive species is critical under the current and future climate scenarios [14]. The information gained will enable the early detection of invasive species and will help establish a rapid response system for controlling and eradicating the invasive species [15,16].

Species distribution models (SDMs) are currently the most reliable techniques used by invasion biologists to investigate the impact of climate change on the geographical distribution and range expansion of invasive species [3,14,17,18]. The SDMs correlate the species occurrence with climatic and other environmental variables, based on the principle of niche conservatism, to generate maps displaying the potential distribution of a given species [19]. Among the various SDMs, the maximum entropy (MaxEnt) model is a popular machine-learning technique that can achieve high predictive accuracy based on a small number of species-occurrence records and environmental variables [20,21]. The algorithm has been widely used to predict the current and future habitats of various invasive species [3,16,22].

The points mentioned above suggest that *P. hysterophorus* is a highly prolific plant under climate change [4] and can be adapted by a variety of environmental factors [23,24]. The average temperature of South Korea has increased by 1.8 °C over the past 100 years, and is predicted to increase by 5.7 °C by 2100 [25]. Therefore, the future climate of South Korea is expected to favor the habitat expansion of *P*. *hysterophorus*. In our earlier studies, we assessed the risk of invasive species, including ecosystem-disturbing alien plants (EDAPs), under the changing climate of South Korea [15,17,26]. The results revealed that South Korea is at a high risk of invasion under future climate conditions. However, because of the limited availability of species-occurrence records, we could not perform the modeling of EDAPs, such as *P. hysterophorus*, in these studies. To the best of our knowledge, no studies have yet been undertaken to reveal the invasion potential of *P. hysterophorus* in South Korea. Therefore, we collected the global occurrence records of *P. hysterophorus* and designed this study. The main objectives of this study were as follows: (1) to predict the current and future distribution range of *P. hysterophorus*, both globally and locally in South Korea, using the MaxEnt algorithm; (2) to evaluate the habitat expansion of *P. hysterophorus* in different administrative divisions (ADs) of South Korea; and (3) to classify the vulnerability of each AD based on the habitat suitability index. Overall, we identified areas at high risk of invasion by *P. hysterophorus*. The results of this study enhance our understanding of the current distribution pattern and future expansion potential of *P*. *hysterophorus* in South Korea and support the construction of a theoretical framework that could be used to develop management strategies for controlling its further spread.

## 2. Materials and Methods

### 2.1. Species-Occurrence Records

**Figure 1:**
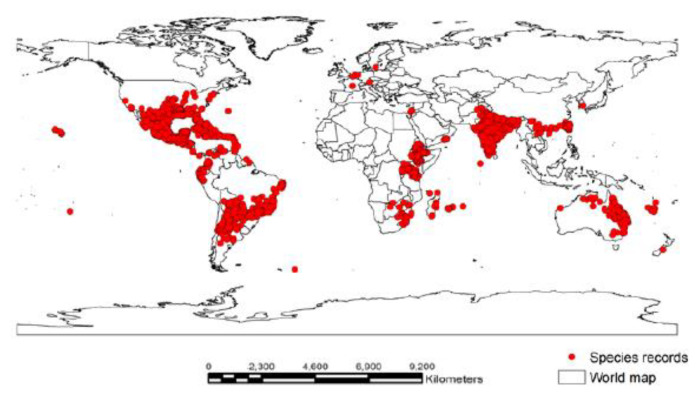
Global occurrence of *P*. *hysterophorus* (*n* = 9234 points). The red dots shown in the figure indicate global positioning points of *P*. *hysterophorus*.

A total of 16,353 global species-occurrence records of *P. hysterophorus* were downloaded from the Global Biodiversity Information Facility [27]. Then, multiple species-occurrence points in the same grid at a spatial resolution of 2.5 min (~4.5 km^2^) were removed and selected a single unique point per grid by using the spatially rarefy occurrence tool in the ArcGIS SDM toolbox v. 2.4 [28]. This process prevents the overfitting and incorrect inflation of the model outcomes, owing to spatial autocorrelation [29]. Finally, the number of species-occurrence points of *P. hysterophorus* was reduced to 9234 (Figure 1 and Table S1). Here, both species-occurrence data sets were used in the MaxEnt modeling of *P. hysterophorus* for investigation of the overestimation of the model.

### 2.2. Selection of Bioclimatic Variables

Nineteen bioclimatic variables, which were considered to be important for predicting the global distribution pattern of *P. hysterophorus*, recorded over a period of 30 years (1970–2000) at a spatial resolution of 2.5 min (~4.5 km at the equator), were downloaded from the WorldClim data portal [30]. WorldClim v2.1 was used to project historical (1970–2000) data, which were considered as the current climatic data. Therefore, these data are hereafter referred to as the current climate data. Similarly, future bioclimatic variables, recorded at the same resolution, were also downloaded from the WorldClim data portal using Coupled Model Intercomparison Project Phase 6 (CMIP6) [31]. The global climate model, Max Planck Institute for Meteorology Earth System Model (MPI-ESM1-2-HR) [32], and two shared socioeconomic pathways (SSPs: SSP2-4.5 and SSP5-8.5) were used to represent the climatic data for the future periods of 2021–2040, 2041–2060, 2061–2080, and 2081–2100. The SSPs are scenarios of projected socioeconomic global changes up to 2100. The SSP scenarios evaluate changes in land use and energy consumption, as well as the corresponding uncertainty in the emission of greenhouse gases and air pollutants [33]. Under SSP2-4.5 and SSP5-8.5, the global mean surface temperature was predicted to increase by 1.8–4.1 K and 3.8–8.6 K, respectively, relative to 1750 [34]. The WorldClim data portal has been used widely for downloading bioclimatic variables for predicting the potential distribution of species in response to the changing temperature and precipitation. The bioclimatic variables serve to delineate and predict the future distribution patterns of species, depending on various climate scenarios and their ecologies [35]. To identify the most important variables for the modeling of *P*. *hysterophorus*, the data of 19 bioclimatic variables downloaded from the WorldClim data portal (Table S2) were subjected to the Spearman’s correlation test. Six variables, including the annual mean temperature (Bio01), mean diurnal temperature range (Bio2), isothermality (Bio03), annual precipitation (Bio12), precipitation in the wettest month (Bio13), and precipitation in the driest month (Bio 14), were ultimately selected for the MaxEnt modeling (Table 1), based on their weak correlation with each other (r < 0.75; Table S3). These six variables were considered as the most important climatic factors for predicting the occurrence of *P*. *hysterophorus*.The Pearson correlation analysis was performed using the PROC CORR function of SAS 9.4 (SAS Institute, Inc., Cary, NC, USA), and six variables were selected, as described previously [36,37] (Table S3). In addition to the bioclimatic variables, other environmental variables, such as land use and landcover change, soil, and human disturbance, could be important variables for determining the distribution of *P*. *hysterophorus*, but future data of such variables are not available under a similar resolution.

### 2.3. Model Development, Evaluation, and Validation

The current and future distribution patterns of *P*. *hysterophorus* were investigated worldwide and in different provinces of South Korea using the MaxEnt Package version 1.33 (http://cran.r-project/org/src/contrib/archive/maxent/ (accessed on 1 September 2022). MaxEnt is a popular machine-learning technique for studying habitat suitability that exhibits high predictive performance based on only a few species-occurrence data points [38]. MaxEnt is generally the best technique for studying invasive species, since the lack of data points for such species may not be reliable as their range may be expanding and may have not reached an equilibrium, which may lead to the misinterpretation of habitat suitability [39]. The background data points of the study area were determined using ArcGIS 10.3, as recommended previously [40], and used for the MaxEnt modeling. In this study, 75% of the species-occurrence data points were used for the model calibration, and the remaining 25% were used for the model validation [41]. The other model options were run with the default settings, and the model was replicated 100 times, as described previously [15].

The goodness-of-fit of the model was measured using three evaluation parameters, namely, the area under the curve (AUC) values of the receiver operating characteristic (ROC) curves [42], the true skill statistic (TSS) [43], and the kappa statistic. When testing model results, the AUC is a threshold-independent method for differentiating between presence and absence. The AUC value, which ranges from 0 to 1, assesses the performance of a model [44]. The AUC value is independent of the size of the dataset (prevalence); however, its use is questionable, because it assigns equal weights to both commission and omission errors, which may prevent accurate predictions [45]. Habitat expansion outside of the species-occurrence range may produce high AUC values, leading to overfitting, a phenomenon that misleads model evaluation [46]. To avoid this problem, other evaluation parameters, such as the TSS and kappa statistics, were also used to understand the accuracy of the model. According to the AUC values, the robustness of the model is rated as failed (0.5–0.6), poor (0.6–0.7), fair (0.7–0.8), good (0.8–0.9), and excellent (0.9–1) [47]. The TSS, which assesses both the specificity and sensitivity of the model, ranges from −1 to +1, accounting for both omission and commission errors [43], and is used as an alternative for assessing the model accuracy [43,48]. The kappa statistic measures the accuracy of the predictions in relation to what may have been discovered by chance alone [43]. Like the TSS, the kappa statistic also ranges from −1 (poor agreement) to +1 (perfect prediction) [43].

### 2.4. Habitat Expansion of P. hysterophorus across the World and in South Korea

The probability distribution map obtained from the MaxEnt modeling was used to produce binary distribution maps of *P. hysterophorus* on a global scale, under the threshold maximum training sensitivity plus specificity cloglog [49], under the current and future climate change scenarios (SSP2-4.5 and SSP5-8.5) for the periods of 2021–2040, 2041–2060, 2061–2080, and 2081–2100. The binary distribution maps show suitable and unsuitable habitats for *P. hysterophorus.* The number of habitat-suitable cells for *P. hysterophorus* were estimated to determine the rate of its habitat expansion on different continents, nations (global scale), and in different ADs of South Korea (local scale) using the Spatial Analyst tool in ArcGIS Desktop 10.8 (Esri, Redlands, CA, USA). The approximate area (1 grid cell = 4.5 km^2^), changes in the habitat suitability, and mean habitat suitability for *P. hysterophorus* were estimated across the different continents, nations, and local ADs in South Korea. Based on the mean suitable value, habitat suitability was classified into four categories, namely, low (≤0.25), moderate (0.26–0.50), high (0.51–0.75), and very high (≥0.76). Similarly, the habitat suitability of 17 ADs in South Korea was classified into the low, moderate, high, and very high categories, based on the mean score of habitat suitability under the current and future climate scenarios.

## 3. Results

### 3.1. Evaluation of Bioclimatic Variables

To investigate the importance of each of the six variables, we estimated their average contribution to the model over five time periods, including the current period (1973–2000) and four future periods (2021–2040, 2041–2060, 2061–2080, and 2081–2100) (Table S4). Among the six variables selected above, three variables (Bio1, Bio3, and Bio13) showed a relatively high contribution to the model. Among these three variables, Bio1 showed the highest average contribution (40.48%), followed by Bio13 (27.19%) and Bio3 (23.75%) (Table 1). Therefore, these three variables were identified as the most prominent factors driving the distribution of *P*. *hysterophorus*; other variables played a relatively smaller contribution in the model’s performance. Similarly, the permutation importance of these bioclimatic variables was estimated, which showed that Bio1, Bio3, and Bio12 are, relatively, the most important variables in the model (Table S5), estimated to be of average importance 35.45%, 21.77%, and 15.18%, respectively. Other variables are relatively less important. The variable importance was further confirmed using the jackknife approach, which estimates the relevance of each variable in the species distribution model and the distinctness of the information provided by each variable. Consistent with the Spearman correlation analysis, the jackknife approach revealed Bio1, Bio3, and Bio13 as the most important variables (Figure S1a–i).

### 3.2. Evaluation of Model Performance Based on AUC, TSS, and Kappa Scores

The model performance was evaluated based on the AUC, TSS, and kappa scores. The AUC, TSS, and kappa scores were highest in the MaxEnt modeling using rarified species-occurrence points compared to the MaxEnt modeling using all the occurrence points (Table 2). It indicates that the model predictions from the rarified species-occurrence points are relatively more accurate and less likely to overestimate the model. Therefore, the model outputs obtained from the rarified occurrence points were selected in this study. The AUC value of the selected MaxEnt model was 0.776, which implies good model performance. Similarly, the TSS and kappa scores were 0.788 and 0.685, respectively, indicating agreement between the observed and predicted data of the model. These results confirmed that the model’s performance when predicting the spatial distribution of *P*. *hysterophorus,* based on the presence-only data, was excellent.

### 3.3. Impact of Climate Change on P. hysterophorus Distribution Worldwide

**Figure 2:**
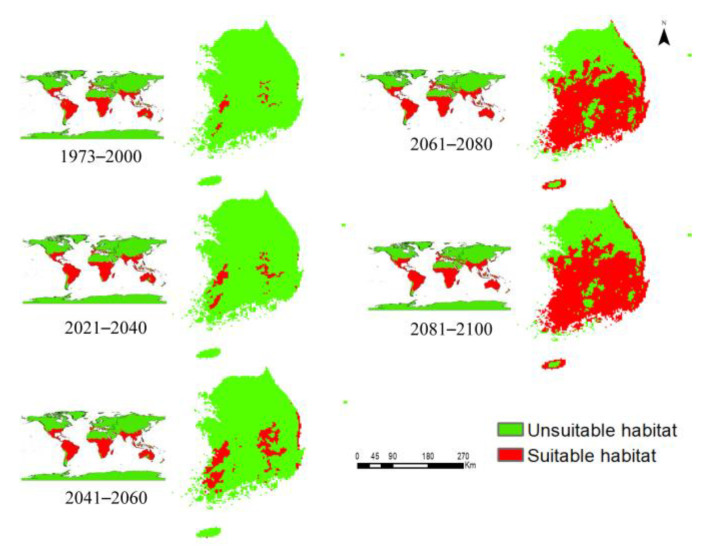
Potential distribution of *P. hysterophorus* around the world and in South Korea under the current climate (1973–2000) and future climate scenario (SSP2-4.5; 2021–2040, 2041–2060, 2061–2080, and 2081–2100). Green and red colors indicate the unsuitable and suitable habitats for *P. hysterophorus*, respectively.

**Figure 3:**
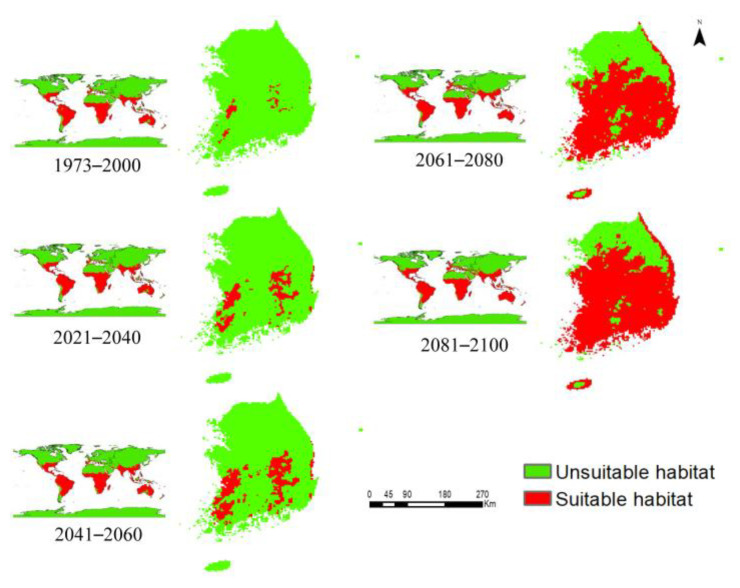
Potential distribution of *P*. *hysterophorus* around the world and in South Korea under the current climate (1973–2000) and future climate scenarios (SSP5-8.5; 2021–2040, 2041–2060, 2061–2080, and 2081–2100). Green and red colors indicate the unsuitable and suitable habitats for *P. hysterophorus*, respectively.

MaxEnt modeling was performed to examine the current and future distribution patterns of *P*. *hysterophorus* under the current climate (1973–2000) and future climate scenarios (SSP2-4.5 and SSP5-8.5; 2021–2040, 2041–2060, 2061–2080, and 2081–2100) (Figure 2 and Figure 3). Under the current climate, *P*. *hysterophorus* shows widespread potential distribution in Australia, South America, Oceania, and Africa, covering approximately 91.26%, 83.26%, 62.75%, and 61.29% of the total land surface of each continent, respectively (Table S6). However, according to the model-based predictions, Europe would be the new invasion hotspot of *P. hysterophorus* in the future. A comparison of future and current habitat suitability indices revealed that the greatest change in *P. hysterophorus* habitat would be observed in Europe (SSP2-4.5= 61.5%, SSP5-8.5 = 56.65%), followed by Oceania (SSP2-4.5 = 34.30%, SSP5-8.5 = 26.76%), and Asia (SSP2-4.5 = 30.92%, SSP5-8.5 = 38.12%), by 2081–2100 (Table 3). These results indicate that the habitat of *P. hysterophorus* would expand in the northern regions of the world, extending up to a latitude of 65°N.

Similarly, the mean habitat suitability for *P. hysterophorus* in all countries around the world was estimated and classified into low, moderate, high, and very high. Under the current climate, 81 countries showed low habitat suitability (Figure S2 and Table S7). However, of these 81 countries, 3 countries (Monaco, Netherlands, and Tokelau (dependent New Zealand territory)) under SSP2-4.5 and 5 countries (Djibouti, Monaco, Netherlands, South Korea, and Tokelau NZ under SSP5-8.5 would transition into the very high suitability category by 2081–2100 (Table 4). These results indicate that climate change would facilitate the expansion of the *P*. *hysterophorus* habitat on the global scale.

### 3.4. Impact of Climate Change on P. hysterophorus Distribution in South Korea

The species distribution model showed that *P*. *hysterophorus* will undergo extreme expansion in South Korea in the future. The current and future distribution patterns of *P*. *hysterophorus* in different time periods are presented in Figure 2 and Figure 3.

**Figure 4:**
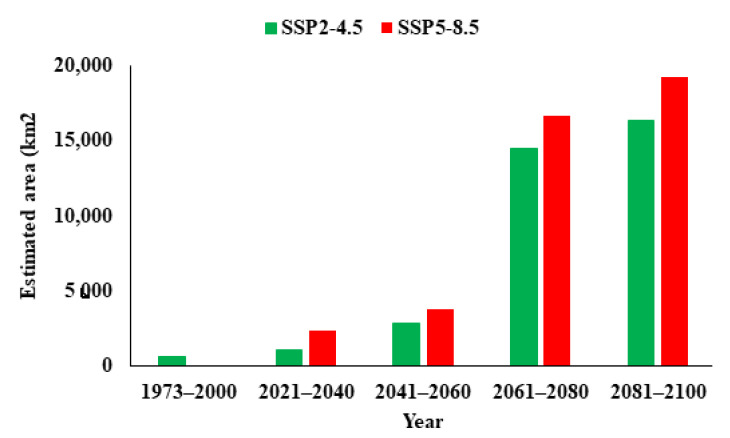
Potential habitat estimation of *P. hysterophorus* in South Korea under the current climate (1973–2000) and future climate scenarios (SSP2-4.5 and SSP5-8.5; 2021–2040, 2041–2060, 2061–2080, and 2081–2100). Green and red bars indicate SSP2-4.5 and SSP5-8.5, respectively.

Based on our model predictions, the current potential distribution of *P*. *hysterophorus* is estimated to be 558 km^2^ in South Korea, covering 2.24% of the total land area of the country (Figure 4). However, in the 2041–2060, 2061–2080, and 2081–2100 periods, the distribution of *P*. *hysterophorus* will increase by 405.64%, 2,491.12%, and 2,835.48%, respectively, under SSP2-4.5, and by 566.93%, 2,883.06%, and 3,343.54%, respectively, under SSP5-8.5. These results suggest that South Korea will be the future hotspot of *P*. *hysterophorus.*

Next, we evaluated the habitat suitability for *P*. *hysterophorus* in the different ADs of South Korea. Table 5 shows the estimated area of *P*. *hysterophorus* under the current and future climate scenarios in 17 ADs. Currently, *P*. *hysterophorus* exists in six ADs, including South Jeolla, North Jeolla, South Chungcheong Gwangju, North Gyeongsang, and Daegue, with each AD estimated to be 18–220.5 km^2^ (Table 5). However, the suitable habitats for *P*. *hysterophorus* would expand across the whole country by 2081–2100 in all ADs (except Incheon and Seoul, located in the northwestern part of the country) under SSP2-4.5 and only in Incheon under SSP5-8.5. By 2081–2100, the abundance of *P*. *hysterophorus* would be the highest in North Gyeongsang (4599 km^2^).

**Figure 5:**
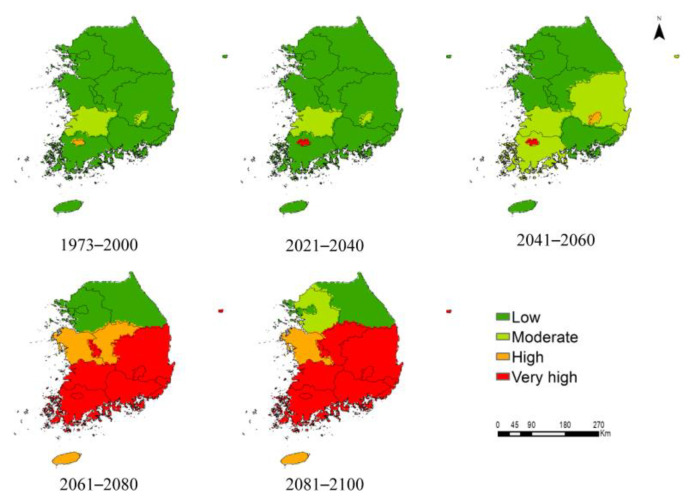
Mean habitat suitability for *P. hysterophorus* in different ADs of South Korea under the current climate (1973–2000) and future climate scenarios (SSP2-4.5; 2021–2040, 2041–2060, 2061–2080, and 2081–2100). The mean habitat suitability for *P. hysterophorus* was classified into four categories (low, moderate, high, and very high), each indicated with a different color.

**Figure 6:**
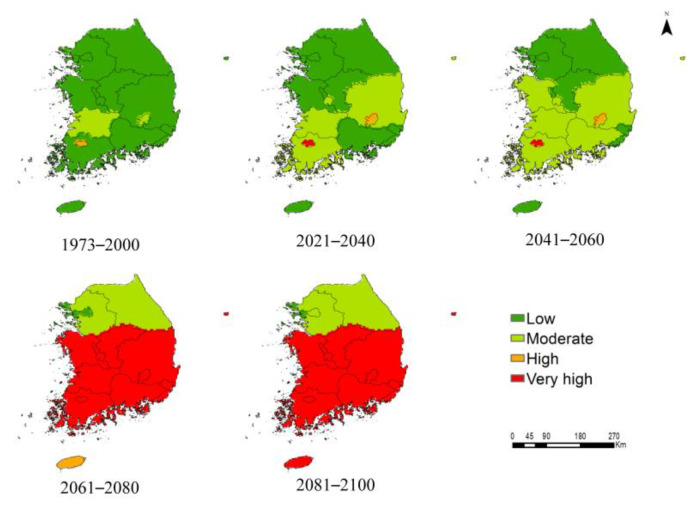
Mean habitat suitability for *P. hysterophorus* in different ADs of South Korea under the current climate (1973–2000) and future climate scenarios (SSP5-8.5; 2021–2040, 2041–2060, 2061-2080, and 2081–2100). Mean habitat suitability was classified into four categories (low, moderate, high, and very high), each of which is indicated with a different color.

The suitable habitats for *P*. *hysterophorus* were classified into four categories (low, moderate, high, and very high), based on the mean habitat suitability measured on a linear scale (Figure 5 and Figure 6). Under the current climate, the habitat suitability for *P*. *hysterophorus* was estimated to be high in Gwangju, and moderate in North Jeolla and Daegue. However, habitat suitability would be high in Jeju and South Chungcheong, and very high in 12 ADs (e.g., South Jeolla, North Chungcheong, and North Gyeongsang) under SSP2-4.5, and very high in 13 ADs under SSP5-8.5 by 2081–2100. These results suggest that climate change will likely promote the northward expansion of *P*. *hysterophorus* habitats in South Korea.

## 4. Discussion

The main findings of this study are as follows: First, among the six bioclimatic variables, the annual mean temperature was the most important variable affecting the global distribution of *P*. *hysterophorus* (Table 1). Second, the model predictions from the rarified species-occurrence points are relatively more accurate and less likely to overestimate the MaxEnt model. Third, under the current climate, Australia showed the highest proportion of suitable habitats relative to its total land area; however, the habitat suitability for *P*. *hysterophorus* is predicted to be the highest in Europe by 2081–2100. Fourth, under the current climate, 81 countries showed low habitat suitability (Table S7); however, under future climate scenarios, five countries, including Djibouti, Monaco, Netherlands, South Korea, and Tokelau (New Zealand), would transition from the low suitability category to the very high suitability category by 2081–2100 (Table 4). Fifth, only 2.24% of the area of South Korea is suitable for *P*. *hysterophorus* under the current climate, but the area covered by *P*. *hysterophorus* is predicted to increase by approximately 3343.54% by 2081–2100 under SSP5-8.5, making South Korea the future hotspot of this invasive species. Lastly, the ADs in the northern and northwestern regions of South Korea are relatively safe from the invasion of *P*. *hysterophorus* compared with those in the southern region of the country (Figure 5 and Figure 6).

The SDM technique is used globally to assess the invasion risk of alien species [50]. Several factors control the performance, and consequently the output, of the model including the size and resolution of the study site, threshold used for modeling [51], type of species, number of species-occurrence records, the type and number of variables selected [52,53], modeling approach [54], global circulation models used [55], methods of model evaluation and validation [46], and thresholds used in binary distribution maps [56]. Among the various algorithms, the MaxEnt model is well known for the modeling of invasive species, because it exhibits high predictive performance using presence-only data [20]; the absence data of invasive species are usually not available, because the ecological ranges of the species may be expanding and may not have reached equilibrium, which may affect the modeling accuracy [39]. Moreover, the MaxEnt model can run easily with many default options [20]. Therefore, we decided to use the MaxEnt model in this study. The predictive performance of this model was tested using the AUC, TSS, and kappa scores, because the use of only the AUC score for the selection of a model is not recommended [43]. In the present study, the AUC, TSS, and kappa scores were calculated as 0.776, 0.788, and 0.685, respectively, which indicated that the model used to study the potential distribution of *P*. *hysterophorus* was robust and highly accurate. Moreover, the MaxEnt model may have overfitting issues due to the sampling bias in geographic and environmental space [29], which could be reduced based on the selection of species-occurrence points. In this study, as a preliminary test, we addressed the issue of overfitting using the rarified species-occurrence points in the model.

The MaxEnt modeling of *P*. *hysterophorus* was performed using six important bioclimatic variables. Among these variables, those related to temperature (Bio1 and Bio3) and precipitation (Bio13) significantly affected the global distribution of *P*. *hysterophorus*. This finding is consistent with some recent studies, which used temperature- and precipitation-related variables as the primary factors determining the distribution of invasive species [3,14,18]. Temperature and precipitation play pivotal roles in plant physiological activities such as germination, growth, and development [57]. *P*. *hysterophorus* can germinate in a wide temperature range (4–36 °C) [23] and can resist drought stress [5]. Moreover, increasing atmospheric CO_2_ concentration increases herbicide, e.g., glyphosate, tolerance capacity and may hinder chemical control effects against *P*. *hysterophorus* [24]. Therefore, future climate change favors the habitat expansion of *P*. *hysterophorus.*

The habitat suitability for *P*. *hysterophorus* not only depends on the bioclimatic variables used in the model but also on many non-climatic factors, including habitat characteristics, land topography, and its superior morphological and physiological characteristics, such as a short life cycle, high fecundity (as evident from the production of 15,000–156,768 viable seeds per specimen [58]), strong dispersal ability, persistent soil seed bank, high germination and growth rates, and high environmental stress tolerance [2]. Therefore, *P*. *hysterophorus* has a strong competitive ability [2,3,4] and can occupy empty niches and substantially expand in new geographical areas [3,14,59,60]. Similarly, many studies showed that disturbed lands, such as roadsides, urban areas, parks, abandoned farms, and orchards, were ideal habitats for *P*. *hysterophorus* [3].

Our study revealed that the global distribution of *P*. *hysterophorus* under the current climate is concentrated in tropical and subtropical regions located in the equatorial belt (35°N to 35°S of the equator), which includes Central America, the Amazon basin of South America, the Congo basin of Africa, and the Indomalayan region of Asia and Australasia. These results are consistent with some recent studies [3,61]. However, by 2081–2100, the global distribution of *P*. *hysterophorus* could expand up to 60°N, and many countries of Europe such as Ireland, the Netherlands, and United Kingdom could become its new hotspots (Table S7). This increase in habitat suitability for *P*. *hysterophorus* in many countries in the Northern Hemisphere could be attributed to climate change [3,4].

Similarly, the mean habitat suitability for *P. hysterophorus* in each country was estimated and classified into four categories (low, moderate, high, and very high) (Figure S2 and Table S7). Among the countries in the low habitat suitability group under the current climate, eight countries, including China, Belgium, and Syria, would transition into the moderate category by 2081–2100; six countries, including Brunei, Bulgaria, and Slovania, into the high habitat suitability category; and five countries, including South Korea, Monaco, and the Netherlands, into the very high habitat suitability group under the SSP5-8.5. However, such changes could not be detected in the global prediction maps because of the high-resolution scale of 2.5 min (~4.5 km^2^) (Figure 2 and Figure 3). The mean habitat suitability was found to be higher under SSP5-8.5 than SSP2-4.5, particularly in the Northern Hemisphere above 30°N latitude. Nonetheless, there were no significant differences from the equator to 15°N latitude (e.g., Colombia, Venezuela, Senegal, Ethiopia, India, and Thailand) or in the Southern Hemisphere (for example, Argentina, Brazil, Zambia, Madagascar, and Australia). This indicates that *P. hysterophorus* can tolerate a wide range of temperatures, and even a low magnitude of warming will allow major expansion of this species. These results are the outcomes of global climate change, increasing the growth and reproduction capacity under elevated CO_2_ levels, higher temperature, and drought [4,11,62], leading to the range expansion of *P*. *hysterophorus*. These results are useful in designing country-specific profiles, depending on the invasion risk category. Furthermore, climate change will create suitable ecological niches for *P*. *hysterophorus* at higher elevations. Although this study did not show the elevational range shift, two separate studies in Bhutan and Nepal showed that the habitat of *P*. *hysterophorus* extends up to 2931 m above sea level (masl) [63,64]. These results suggest that climate change would facilitate the habitat expansion of *P*. *hysterophorus* to higher altitudes and latitudes, owing to the increase in temperature.

In South Korea, our model predicted that small areas of the country are climatically suitable for *P*. *hysterophorus* under the current conditions. Total suitable habitat area was estimated under the current conditions. Our model predicted that a total of 558 km^2^ in six ADs would be invaded by *P*. *hysterophorus* under the current climate. Three of these six ADs including North Jeolla, South Jeolla, and North Gyeongsang, which are located in the western, southern, and eastern regions of South Korea, respectively, have been invaded to a greater extent than the other three ADs. All of the invaded ADs of South Korea are located below 36°N, and these regions are characterized by a warm temperate climate with high humidity [25]. These conditions may favor the establishment of not only *P*. *hysterophorus* but also many other invasive weeds found in the tropical and subtropical regions of the Americas, Southern Europe, and East and South Asia [1,2,10]. The invasive weeds native to tropical and subtropical climates exhibit relatively higher critical thermal maxima than indigenous species, suggesting that such invasive species can flourish at higher temperatures and can dominate the indigenous species under the changing climate [60].

Under the future climate, the invasion by *P*. *hysterophorus* would substantially increase and expand northward. By 2081–2100, the *P*. *hysterophorus* habitat would expand in all the ADs of South Korea, except Seoul and Incheon, which are located above 37°N in the northwestern region of the country. These results complement the spatially explicit evidence, thus supporting the earlier hypothesis, according to which increasing temperatures are expected to expand invasion threats in the northward direction [65], consistent with previous reports [15,63,64,66]. Moreover, our study showed that the ADs present in the southern and central regions of South Korea, except Gwangju, with a mean habitat suitability ranging from low to high in 2041–2060, would advance to the very high suitability category by 2081–2100 (Figure 6). With the rising global temperature, the habitat suitability for *P*. *hysterophorus* is predicted to expand towards the central and northern regions, similar to that of other invasive species in South Korea [15,16,22], because of the elimination of current climatic barriers, thus impelling the establishment of plant hardiness zones northward [60,67].

Besides climate change, anthropogenic factors such as the construction of roads and railway networks could facilitate the invasion of adjacent crop fields and pastures [68]. In South Korea, many highways and railroads connect the north to the south, which may assist in the habitat expansion of *P*. *hysterophorus.* Similarly, natural phenomena, such as typhoon, wind, water, and wild animals, as well as human activities such as the import of contaminated grains, vegetables, and pasture seeds may promote invasion [4]. South Korea also experiences such natural phenomena and human activities, and therefore is not immune to invasion by *P*. *hysterophorus* in the future.

*P*. *hysterophorus* has detrimental impacts on agricultural and natural ecosystems by decreasing crop yield, degrading pastures, and reducing the forage of livestock and wild herbivores (e.g., roe deer [69]), and threatening forest ecosystems [70]. Agricultural land in the western, southern, and eastern regions of South Korea are under high risk of invasion of *P*. *hysterophorus*, which may cause high economic losses and negatively impact food security, native biodiversity, and ecosystem services in the country. Therefore, strict quarantine measures are required to limit the habitat expansion of *P*. *hysterophorus,* both globally and locally. Additionally, policymakers, land resource managers, and local stakeholders need to develop invasion control strategies to prevent further invasions.

## 5. Conclusions

*P*. *hysterophorus* is a major threat to agriculture, biodiversity, natural ecosystems, and, consequently, to the national economy. To minimize the negative impact of *P*. *hysterophorus*, we selected the MaxEnt model to predict its potential distribution around the world and in South Korea. Our findings suggest that *P*. *hysterophorus* is distributed between 35°N and 35°S of the equator under the current climate, and its distribution is relatively high in Australia, South America, Oceania, and Africa. However, under future climate scenarios (SSP2-4.5 and SSP5-8.5), the habitat of *P*. *hysterophorus* would expand extensively (up to 65° N of the equator), while retaining its current distribution range, and Europe would be the new invasion hotspot. In South Korea, a small portion of the country (2.24%) is predicted to be invaded by *P*. *hysterophorus*, particularly in six ADs (mainly North Jeolla, South Jeolla, and North Gyeongsang) under the current climate. However, in the future, the habitat of *P*. *hysterophorus* would expand in all ADs, except Incheon, which is located in the northwestern part of the country. Similarly, an estimation of the mean habitat suitability revealed that the central and southern regions of the country would be very highly suitable for *P*. *hysterophorus* invasion. Therefore, careful planning and long-term management strategies are needed at the global, national, and local levels to reduce the habitat expansion of this noxious weed. The MaxEnt modeling approach can assist the government in prioritizing the geographical locations for early detection and eradication and can suggest the best preventive measures.

## Figures and Tables

**Table 1:** Bioclimatic variables selected for modeling *P*. *hysterophorus* distribution.

Code	Description	Unit	Model Contribution (%) ^1^
Bio1	Annual mean temperature	°C	40.48
Bio2	Mean diurnal temperature range	°C	0.40
Bio3	Isothermality (BIO2/BIO7) (×100)	%	23.75
Bio12	Annual precipitation	mm	2.00
Bio13	Precipitation in the wettest month	mm	27.19
Bio14	Precipitation in the driest month	mm	5.85

^1^ Model contributions represent the average values of nine models established under the current and future climate change scenarios (SSP2-4.5 and SSP5-8.5) for five designated periods (1973–2000, 2021–2040, 2041–2060, 2061–2080, and 2081–2100).

**Table 2:** Evaluation parameters of MaxEnt modeling of *P*. *hysterophorus*.

Evaluation Parameter	Before Rarifying	After Rarifying
Species occurrence points	16,353	9234
AUC	0.612	0.776
TSS	0.624	0.788
Kappa	0.513	0.685

**Table 3:** Change in the suitable habitat (%) of *P. hysterophorus* in different continents in future compared to the current climatic condition (1973–2000).

Continent	1973–2000 (km^2^)	SSP2-4.5 ^1^				SSP5-8.5 ^2^			
		2021–2040	2041–2060	2061–2080	2081–2100	2021–2040	2041–2060	2061–2080	2081–2100
Africa	4,063,351.5	−0.28	−16.59	7.92	8.75	1.11	0.13	10.04	10.94
Antarctica	0	0	0	0	0	0	0	0	0
Asia	2,225,502	2.82	8.33	28.24	30.92	8.27	11.89	32.29	38.12
Australia	1,641,442.5	5.52	−1.41	9.03	9.29	0.24	−3.37	8.88	9.29
Europe	463,756.5	−13.55	34.49	53.18	61.05	−8.59	−26.40	71.78	56.65
North America	1,416,852	1.58	0.96	17.73	20.14	−0.29	0.64	17.86	22.05
Oceania	55,350	7.13	10.82	32.21	34.30	−0.78	−7.79	28.15	26.76
South America	3,309,093	−0.89	−6.05	5.15	5.68	1.80	1.83	5.24	5.66

^1,2^ Minus (−) sign indicates a decrease in the suitable habitat area of *P. hysterophorus*.

**Table 4:** Predicted habitat suitability for *P*. *hysterophorus* in different nations in 2081–2100 under future climate scenarios (SSP2-4.5 and SSP5-8.5).

Habitat Suitability ^1^	SSP2-4.5	SSP5-8.5
Moderate	Brunei, Chile, China, Georgia, Iran, Japan, Liechtenstein, Montenegro, Niger, Syria, Yemen	Belgium, China, Georgia, Iran, Japan, Niger, Syria, Yemen
High	Belgium, Bulgaria, Djibouti, Slovenia, South Korea	Brunei, Bulgaria, Liechtenstein, Macedonia, Montenegro, Slovenia
Very high	Monaco, Netherlands, Tokelau (New Zealand)	Djibouti, Monaco, Netherlands, South Korea, Tokelau (New Zealand)

^1^ Moderate habitat suitability (0.25–0.50); high habitat suitability (0.51–0.75); and very high habitat suitability (0.76–1). Here, some countries with low habitat suitability (≤0.25) in 1973–2000 are predicted to transition into the moderate, high, and very high habitat suitability categories by 2081–2100 under the future climate scenarios SSP2-4.5 and SSP5-8.5. The habtiat suitability value of each category is presented in Table S7.

**Table 5:** Estimation of suitable habitat for *P. hysterophorus* in the different ADs of South Korea under the current climate (1973–2000) and future climate scenarios (SSP2-4.5 and SSP5-8.5; 2021–2040, 2041–2060, 2061–2080, and 2081–2100).

AD	Total Area (km^2^)	1973–2000	SSP2-4.5				SSP5-8.5			
			2021–2040	2041–2060	2061–2080	2081–2100	2021–2040	2041–2060	2061–2080	2081–2100
Busan	193.5	0	0	0	162	180	0	0	184.5	189
North Chungcheong	1926	0	0	0	1161	1453.5	0	22.5	1624.5	1773
South Chungcheong	2115	18	45	81	1264.5	1458	99	225	1593	1993.5
Daegu	220.5	31.5	85.5	108	198	207	112.5	130.5	207	216
Daejeon	144	0	0	4.5	144	144	18	54	144	144
Gangwon	4392	0	0	13.5	283.5	418.5	0	0	504	886.5
Gwangju	130.5	54	63	117	130.5	130.5	94.5	117	130.5	130.5
Gyeonggi	2695.5	0	0	0	193.5	306	0	0	378	1057.5
North Gyeongsang	4963.5	193.5	333	1152	3838.5	4140	972	1381.5	4351.5	4599
South Gyeongsang	2709	0	18	135	2178	2358	144	378	2331	2524.5
Incheon	261	0	0	0	0	0	0	0	0	0
Jeju	459	0	0	4.5	292.5	310.5	0	0	301.5	337.5
North Jeolla	2052	220.5	369	648	1737	1813.5	594	796.5	1836	1944
South Jeolla	3055.5	40.5	157.5	504	2484	2664	306	580.5	2637	2907
Sejong	117	0	0	0	99	117	0	0	117	117
Seoul	148.5	0	0	0	0	0	0	0	0	36
Ulsan	274.5	0	13.5	45	234	243	27	27	243	256.5

## Data Availability

Not applicable.

## Supplementary Materials

The following supporting information can be downloaded at: https://www.mdpi.com/article/10.3390/biology12010084/s1, Table S1: Global occurrence points of *P. hysterophorus*; Table S2: List of bioclimatic variables; Table S3: Estimation of Pearson correlation coefficient for selecting bioclimatic variables; Table S4: Contribution of different bioclimatic variables in model performance corresponding to different time periods; Table S5: Permutation importance of different bioclimatic variables in model; Table S6: Estimation of suitable habitat area (km^2^) of *P. hysterophorus* in different continents under the current (1973–2000) and future climate scenarios SSP2-4.5 and SSP5-8.5; Table S7: Estimation of approximate area of suitable habitat (km^2^) and mean habitat suitability for *P*. *hysterophorus* in different nations of the world under the current climate and future climate scenarios, SSP2-4.5 and SSP5-8.5; Figure S1: Jackknife test-based analysis of the model used to predict the habitat expansion of *P. hysterophorus* under the current climate (1973–2000) and future climate scenarios (SSP2-4.5 and SSP5-8.5; 2021–2040, 2041–2060, 2061-2080, and 2081–2100). Figure S2: Estimation of the mean habitat suitability for *P. hysterophorus* in different countries of the world under the current climate (1973–2000) and future climate scenarios (SSP2-4.5 and SSP5; 2021–2040, 2041–2060, 2061–2080, and 2081–2100).
