# Supplementary material for: Predicting the Impact of Climate Change on the Habitat Distribution of Parthenium hysterophorus around the World and in South Korea
Source: Biology (Basel). 2023 Jan 4;12(1):84. doi: 10.3390/biology12010084 (PMC9855788; doi:10.3390/biology12010084)
Supplement: Supplementary file 1 [file biology-12-00084-s001.zip › Figure S1.pdf]

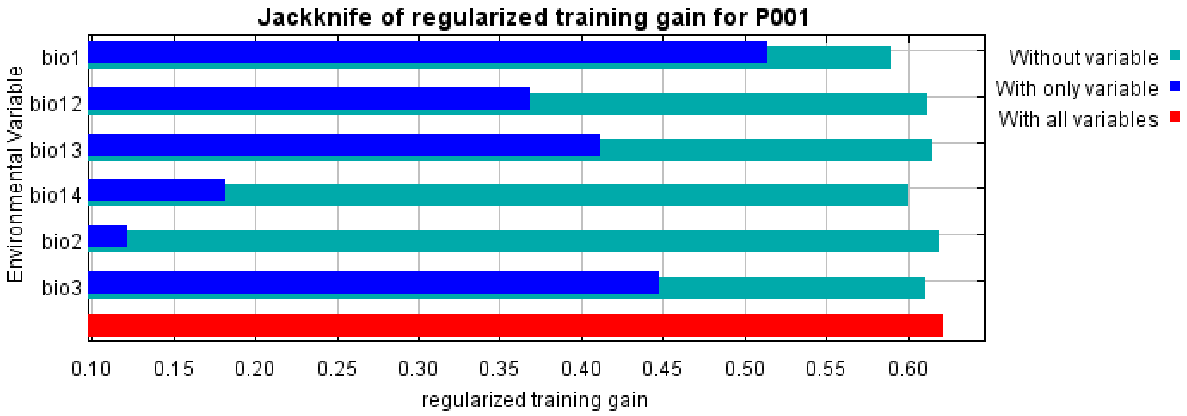

Figure S1a. Jackknife test-based analysis of the model used to predict the habitat expansion of *P. hysterophorus* under the current climate (1973-2000).

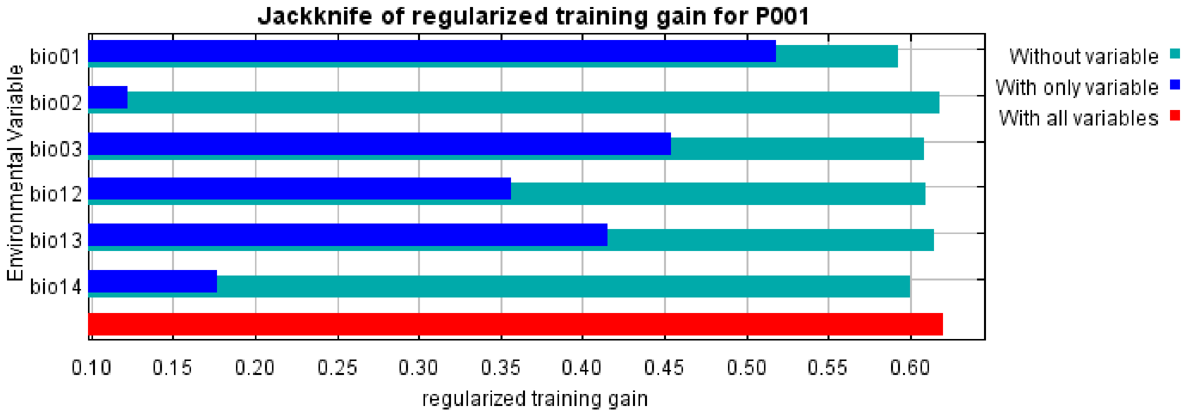

Figure S1b. Jackknife test-based analysis of the model used to predict the habitat expansion of *P. hysterophorus* under the climate change scenario SSP245 for the time period 2021 to 2040.

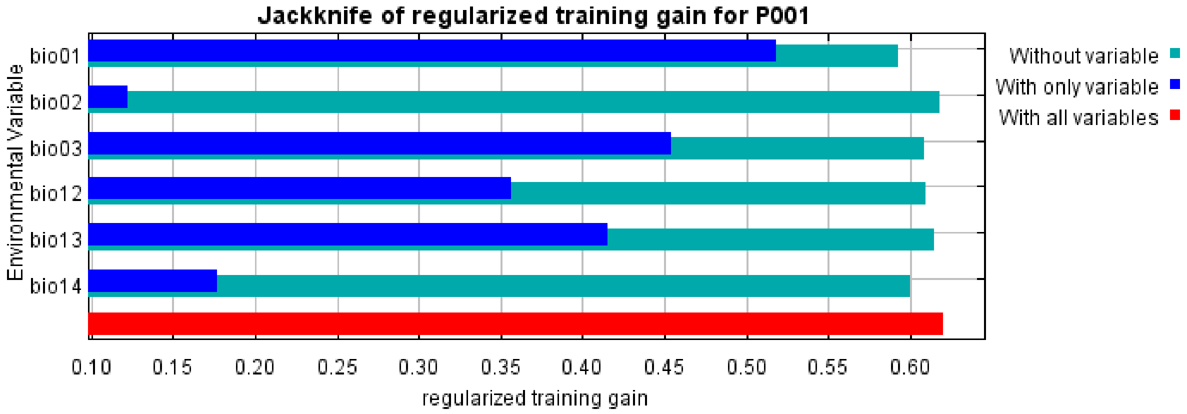

Figure S1c. Jackknife test-based analysis of the model used to predict the habitat expansion of *P. hysterophorus* under the climate change scenario SSP245 for the time period 2041 to 2060.

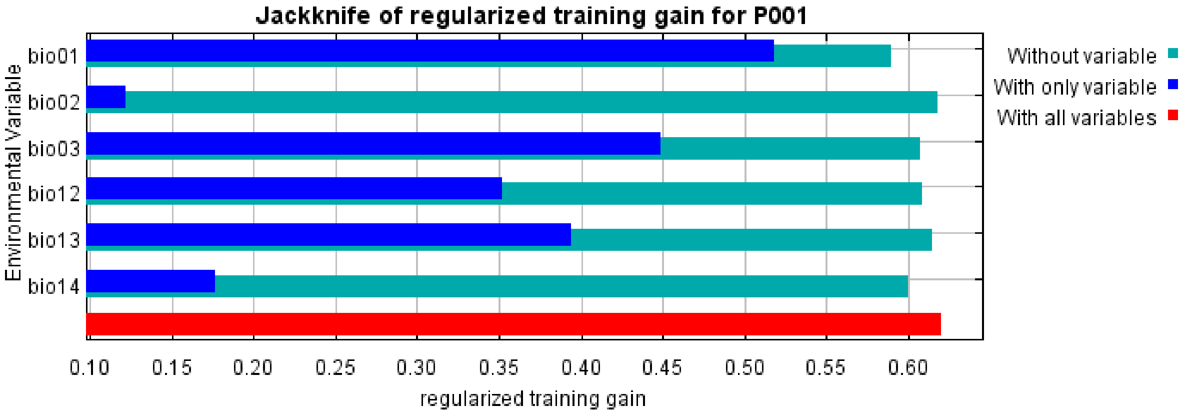

Figure S1d. Jackknife test-based analysis of the model used to predict the habitat expansion of *P. hysterophorus* under the climate change scenario SSP245 for the time period 2061 to 2080.

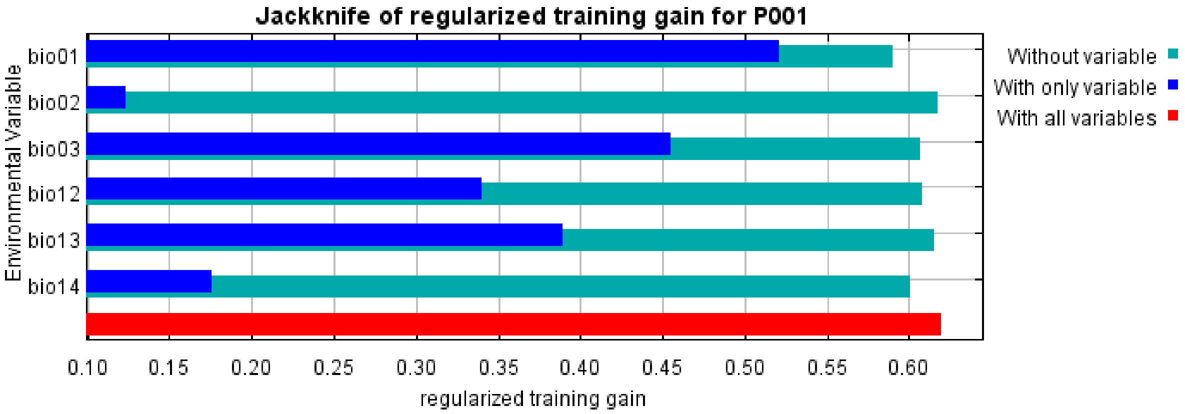

Figure S1e. Jackknife test-based analysis of the model used to predict the habitat expansion of *P. hysterophorus* under the climate change scenario SSP245 for the time period 2081 to 2100.

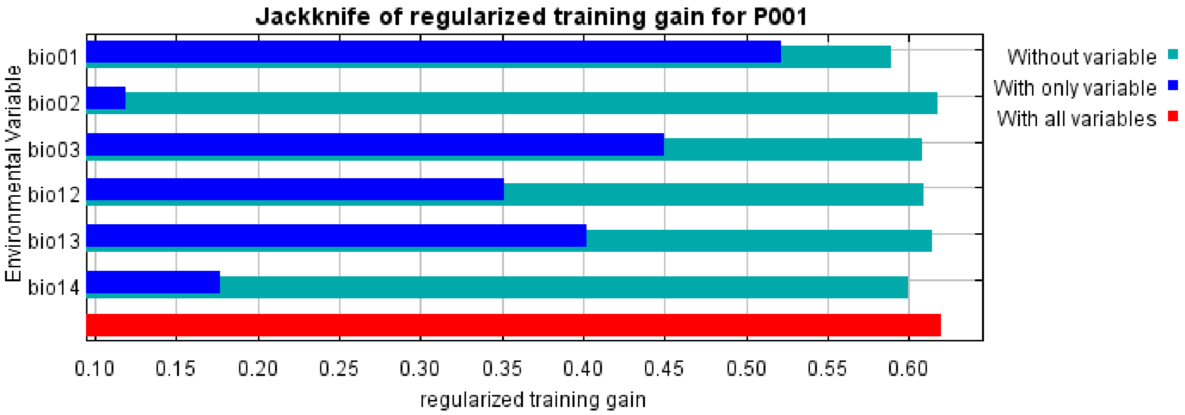

Figure S1f. Jackknife test-based analysis of the model used to predict the habitat expansion of *P. hysterophorus* under the climate change scenario SSP585 for the time period 2021 to 2040.

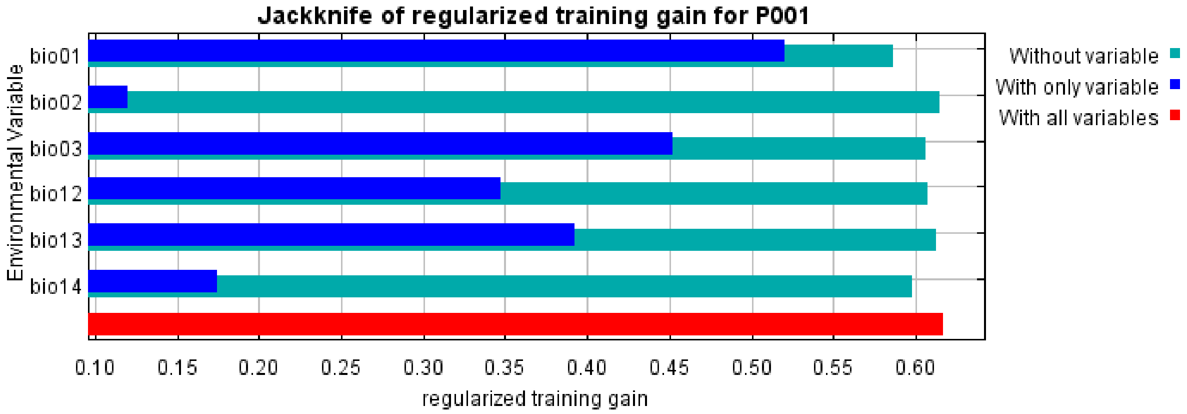

Figure S1g. Jackknife test-based analysis of the model used to predict the habitat expansion of *P. hysterophorus* under the climate change scenario SSP585 for the time period 2041 to 2060.

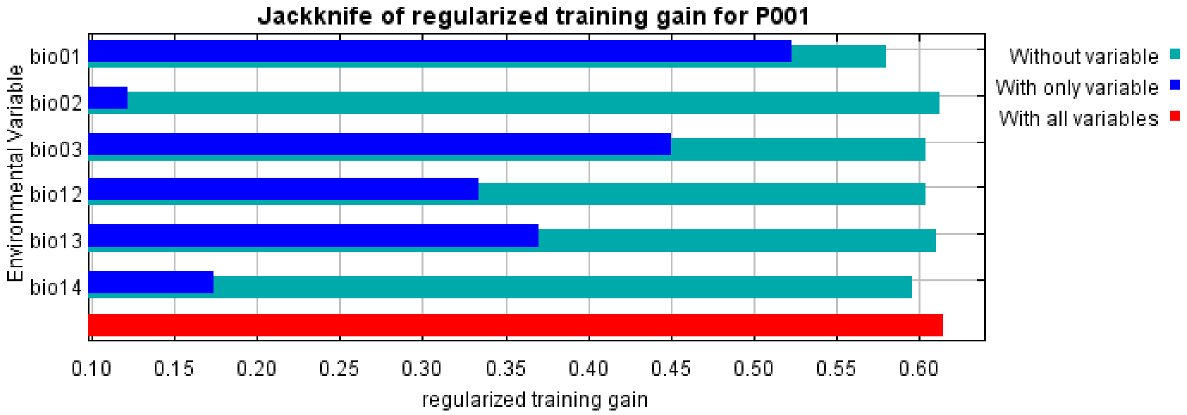

Figure S1h. Jackknife test-based analysis of the model used to predict the habitat expansion of *P. hysterophorus* under the climate change scenario SSP585 for the time period 2061 to 2080.

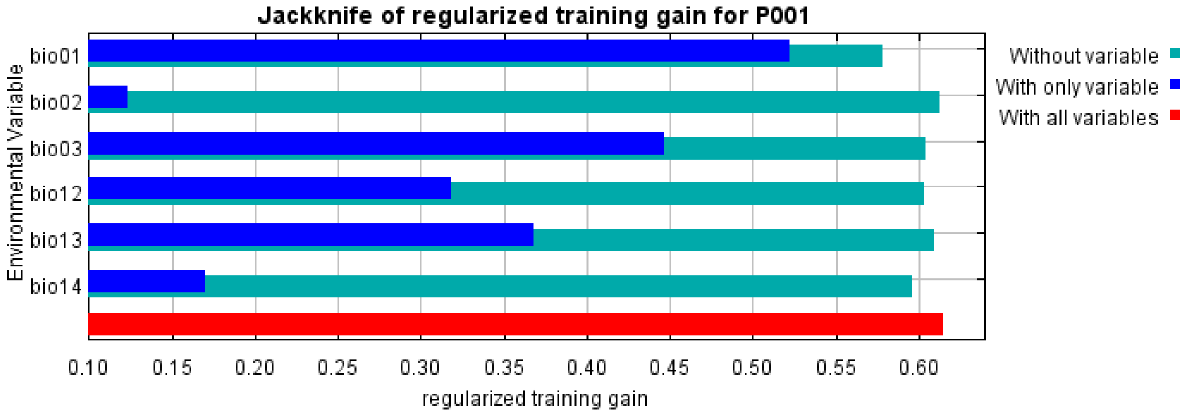

Figure S1i. Jackknife test-based analysis of the model used to predict the habitat expansion of *P. hysterophorus* under the climate change scenario SSP585 for the time period 2081 to 22100.
