# Supplementary material for: Predicting the Impact of Climate Change on the Habitat Distribution of Parthenium hysterophorus around the World and in South Korea
Source: Biology (Basel). 2023 Jan 4;12(1):84. doi: 10.3390/biology12010084 (PMC9855788; doi:10.3390/biology12010084)
Supplement: Supplementary file 1 [file biology-12-00084-s001.zip › Figure S2.pdf]

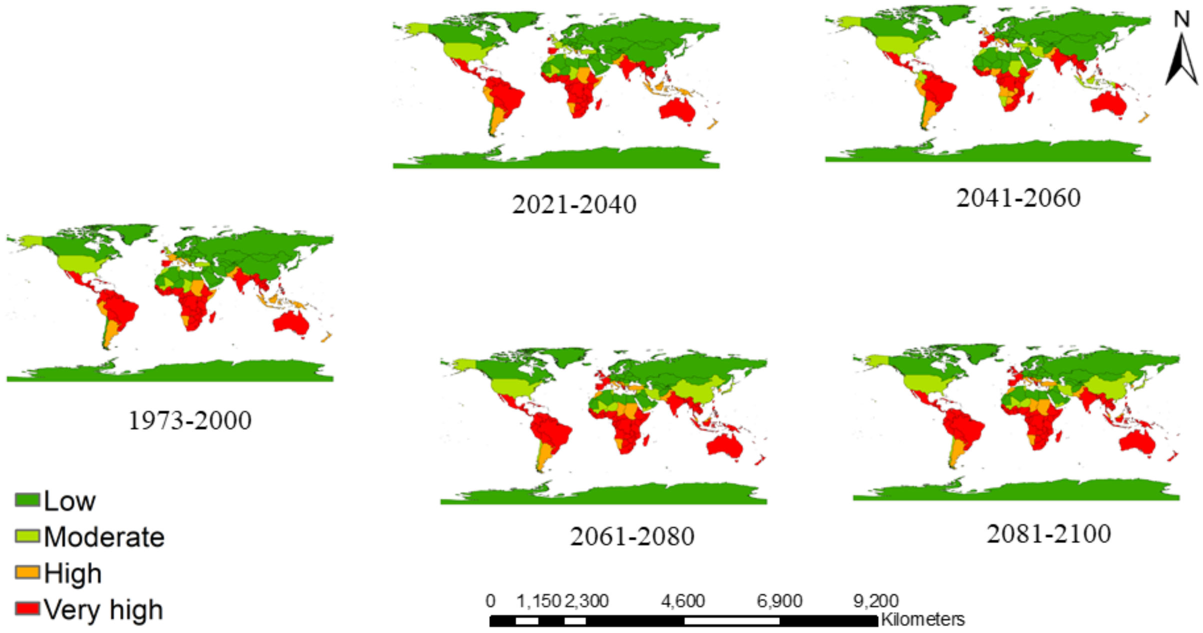

Figure S2a. Estimation of the mean habitat suitability of *P. hysterophorus* in different countries of the world under the current climate (1973–2000) and future climate scenario (SSP2-4.5; 2021–2040, 2041–2060, 2061-2080, and 2081–2100). Mean habitat suitability was classified into four categories (low, moderate, high, and very high), each of which is indicated with a different color.

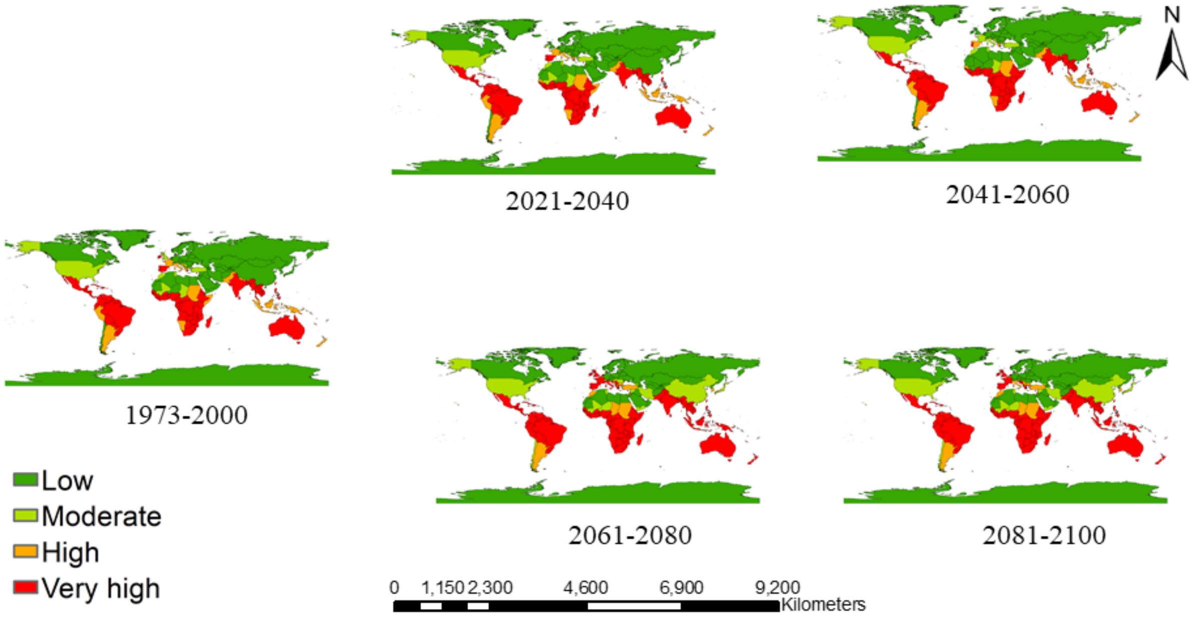

Figure S2b. Estimation of the mean habitat suitability of *P. hysterophorus* in different countries of the world under the current climate (1973–2000) and future climate scenario (SSP5-8.5; 2021–2040, 2041–2060, 2061-2080, and 2081–2100). Mean habitat suitability was classified into four categories (low, moderate, high, and very high), each of which is indicated with a different color.
